# Supplementary material for: YAP controls retinal stem cell DNA replication timing and genomic stability
Source: eLife. 2015 Sep 22;4:e08488. doi: 10.7554/eLife.08488 (PMC4578106; doi:10.7554/eLife.08488)
Supplement: Supplementary file 1. — Sequences of Morpholino oligonucleotides and primers used in the study. DOI: http://dx.doi.org/10.7554/eLife.08488.019 [file elife08488s001.docx]

**Supplementary file 1**

|  | MORPHOLINOS SEQUENCE |
| --- | --- |
| *Yap-*MO | TAGGAGACTGTGGGTCACTTCACCA-Lissamine |
| *Yap*-MO with 5 mismatch | TAGCAGAGTGTGGCTCAGTTCACGA-Lissamine |
| *pknox1*-MO | ATGATACACTGCCCCTTGGCCTTTC-Lissamine |
| *pknox1*-MO with 5 mismatch | ATCATAGACTGCCCGTTGCCCTTTG-Lissamine |
| *Yap-*AS-photo-MO | TAGGAGACTGTGPGTCACTTCACC |
| *Yap-*S-photo-MO | TGAAGTGACPCACAGTCTCC |
| Standard control-MO | CCTCTTACCTCAGTTACAATTTATA |

|  | qPCR PRIMER SEQUENCES |
| --- | --- |
| *c-Myc* forward | GGCGGAACGAGCTTAAGTTG |
| *c-Myc* reverse | CGCCACCTCGGGTACCT |
| *ODC* forward | GCTTCTGGAGCGGGCAAAGGA |
| *ODC* reverse | CCAAGCTCAGCCCCCATGTCA |
| *RPL8* forward | CCACGTGTCCGTGGTGTGGCTA |
| *RPL8* reverse | GCGCAGACGACCAGTACGACGA |

|  | cloning PRIMER SEQUENCES |
| --- | --- |
| *Yap* forward | GAATTCGGAGCCCGGCTCCCAGCAACAACCTTCC |
| *Yap* reverse | CTCGAGTAACCACGTGAGGAAGCTTTCCTTGT |
| *Pknox1* forward | GAATTCAATGATGTCTGCTGAGAGACTGGAC |
| *Pknox1* reverse (myc-pCS2+) | CTCGAGAGCTGGAGCCCCCCCACTCAGCACTAGTCCCCTCA |
| *Pknox1* reverse (HA-pCS2+) | GAATTCATGATGTCTGCTGAGAGACTGGAC |
| *Tead1* forward | AGGCCTTAATGGAGCCGAGCAGTTG |
| *Tead1* reverse | TCTAGATCTTTAACAAGCCTGTAGATGTGATGC |
| *p53* forward | CGGAATTCCATGGAACCTTCCTCTGAGACCGGC |
| *p53* reverse | GCTCTAGATCATTCCGAGTCGGGCTGTTCATC |
