# Supplementary material for: YAP controls retinal stem cell DNA replication timing and genomic stability
Source: eLife. 2015 Sep 22;4:e08488. doi: 10.7554/eLife.08488 (PMC4578106; doi:10.7554/eLife.08488)
Supplement: Supplementary file 2. — List of antibodies used in the study. DOI: http://dx.doi.org/10.7554/eLife.08488.020 [file elife08488s002.docx]

**Supplementary file 2**

| ANTIBODIES | DILUTION | COMPANY | TECHNIQUE |
| --- | --- | --- | --- |
| Primary mouse monoclonal antibodies | | | |
| anti-H2AX | 1:50 | Millipore | retina IF ^(a)(b)^ |
| anti-HA | 1:300 | Sigma | HEK293T IF |
| anti-myc | 1:200 | Invitrogen | HEK293T IF |
| anti-PCNA | 1:1000 | Dako | retina IF ^(c)^ |
| anti-YAP | 1:50 | Abcam | retina IF ^(a)^ |
| Primary rabbit polyclonal antibodies | | | |
| anti-Flag | 1:300 / 1:4000 | Sigma | HEK293T IF / HEK293T WB |
| anti-HA | 1:4000 | Sigma | HEK293T WB |
| anti-PH3 | 1:500 | Millipore | retina IF |
| anti-YAP | 1:1000 | Santa Cruz Biotechnology | Xenopus WB |
| anti-α-tubulin | 1:1000 | Abcam | Xenopus WB |
| Secondary goat antibodies | | | |
| Alexa 448 Anti-mouse or rabbit | 1:1000 | Molecular Probes | retina IF |
| Alexa 546 Anti-mouse or rabbit | 1:1000 | Molecular Probes | HEK293T IF |
| Alexa 647 Anti-mouse or rabbit | 1:1000 | Molecular Probes | HEK293T and retina IF |
| Alexa 694 Anti-mouse or rabbit | 1:1000 | Molecular Probes | retina IF |

^(a)^ Amplification using M.O.M Immunodetection kit (Vector Laboratories). ^(b)^ Antigen unmasking by 5 min incubation in 2N HCl solution and 20 µg/ml RNase treatment. ^(c)^ Fixation in Bouin solution and antigen unmasking by 9 min heating in a boiling 10mM sodium citrate + 0.05% tween solution followed by 45 min incubation in 2N HCl solution. IF: Immunofluorescence; WB: Western blot.
