# Supplementary material for: YAP controls retinal stem cell DNA replication timing and genomic stability
Source: eLife. 2015 Sep 22;4:e08488. doi: 10.7554/eLife.08488 (PMC4578106; doi:10.7554/eLife.08488)
Supplement: Supplementary file 3. — Sequences used in the NanoString experiment. DOI: http://dx.doi.org/10.7554/eLife.08488.021 [file elife08488s003.docx]

**Supplementary file 3.**

| TARGET | ACCESSION | TARGET REGION | TARGET SEQUENCE |
| --- | --- | --- | --- |
| Cell cycle genes | | | |
| cdc-2 | NM_001086624.1 | 875-975 | GAGGATTTCCGCACGAAAAGCTATGTTGCACCCCTACTTCGATGACTTGGATAAGTCCAGCCTTCCCGCCAATCAGATTAGAAATTAAAACGGCAAATGT |
| cdc25c-a | NM_001087787.1 | 2297-2397 | AAGCAGAGAAAAAAACCTAAACCCACTCAGTCTGCAAGCTCCAGAACTAACTTAGAGAATGGGCAGAATAACTGCGCTGCAGTTGAGACAACATGTAATA |
| cdk2 | NM_001090651.1 | 235-335 | CATACGGAAAACAAGCTCTACCTTGTCTTCGAGTTCCTTAACCAAGATCTGAAGAAATTCATGGACCGGTCAAACATTTCTGGAATTTCATTGGCCCTAG |
| cyclin A2 | NM_001096243.1 | 61-161 | ATCGGTGTGCACGGTATGGCCGACCCTCTGTTGCGGGACGAGAATCAGGAAAATGTTCAGCCCCGAAAGCCCCTCGCCCCTGTGGGTGGACGCACCGTGC |
| cyclin B1 | NM_001088520.1 | 854-954 | AATTGGAGAGGTTGACTCTGTACATCACACACTGGCTAAATACCTAATCGAGCTGGTTATGACAGACTATGACATGGTGCACATACCTCCATCACAGTTG |
| cyclin D1 | NM_001086776.1 | 904-1004 | AAAAACATGGTGGATGAAGTGGACATCTCCTGCACCCCGACCGATGTCCGAGACGTCAACATCTGACATCAAACGGATCACAGACATTTTTTTTTGCTCA |
| cyclin D2 | NM_001096348.1 | 49-149 | ATAAGCGCAGGGAGCAGCAATAAGGCTCAGCGGCAGAGTGGGGCTCAACTACTATCCCGGCAACGGGGGGAGGGGAATCATCCAGTGGTAGCGCCCAAGG |
| cyclinE | NM_001087977.1 | 35-135 | CTCGCCTCGTCACATCTGCCGCTGCTCTTGTCCGTCTCCTTTCGCCATTGGATCCGGTGCGTGGGACAGAAAAGTTACCCTTCATTCATTACAAAGCCCT |
| ki67 | NM_001135081.1 | 2812-2912 | CAGGACATGCAGATTCTCCTGCCACAATCCTTGTTGGAAGAGCTCATACAAGAACGGTCAACCCAACTGGATATGTTCCTAAAATTGTGCAAAACCAAGC |
| p19 | NM_001093415.1 | 787-887 | TATAGTTCAGCATGTTTGTTACTGACTGCGGAGAGCTGCAATAGTACCAATGCCCTATATATTGTTCTAAATACTGGGAACAACTGCAGCACCCTTCAAG |
| p21Xic2 | AY573848.1 | 346-446 | CCAGAATTGTGAAAGCAGCGATTCAGGCAAACGAAAACAAAAGCTGATCACAGATTTTTATCCTGTCAAGCGGAGATGTTCACCCGTGCCCAGCCTCCAT |
| p27Xic1 | NM_001088319.1 | 816-916 | TACCGAGATCCGTGGCTCCGGGGACTGCTGAATCCACGGGGTTATTCGTTTCTACTTGGTATCTGGAGATTGTGGCAATAAGGGACTTGTTTCGTGTGCC |
| p53 | NM_001088098.1 | 1781-1881 | CATCTGTGTGTGGGACAACCAGTTATTTATTTGGGCTAGCTGCAGGCTTTTTATTATGTGACCCTATTTGTGAGGATATGAATGAGCCCTGCTGTGGCCA |
| pcna | NM_001087542.1 | 812-912 | GAAGCTTCTTAATGTCTGAACTAGCTTATTTTATAAACCTCAACTGAACGTCCAATGGCGCTTTCACACACCTGCCTTGTTTTAACAGCTTTGGCTGAAC |
| Xic3 | AY573849.1 | 117-217 | TTGGCCCGGGACTTTGAGCGGAGCCTTCGGGCGATGGAGGAAGAGAAACGCGAGAAGTGGAACTTCGACTTCCGCAACTACCGGCCGCTACCGGGGCCCC |
| House keeping genes | | | |
| gapdh | NM_001087098.1 | 773-873 | ACCTGCCGCCTGCAGAAGCCGGCCAAGTACGATGACATCAAGGCCGCCATTAAGACTGCATCAGAGGGCCCAATGAAGGGAATCCTGGGATACACACAAG |
| odc | NM_001086698.1 | 855-955 | GGATATAATTGGTGTGAGTTTCCATGTTGGCAGTGGCTGCACTGATCCACAGACTTATGTACAAGCTGTCTCAGATGCACGATGTGTCTTTGACATGGGG |
| H4 | NM_001094457.1 | 129-229 | GGAGAGGGGGAGTCAAGCGCATCTCTGGCCTCATCTATGAGGAGACTCGTGGGGTCCTCAAGGTTTTCCTGGAGAATGTCATCCGGGACGCCGTCACCTA |
